# Parental psychosocial factors, unmet dental needs and preventive dental care in children and adolescents with special health care needs: A stress process model

**DOI:** 10.1186/s12903-022-02314-y

**Published:** 2022-07-11

**Authors:** Arwa Z. Gazzaz, Richard M. Carpiano, Denise M. Laronde, Jolanta Aleksejuniene

**Affiliations:** 1 Department of Oral Health Science, Faculty of Dentistry, University of British Columbia, Vancouver, BC Canada; 2 Department of Periodontics and Community Dentistry, College of Dentistry, King Saud University, Riyadh, Saudi Arabia; 3 School of Public Policy, University of California, Riverside, CA USA; 4 Department of Oral Biological and Medical Sciences, Faculty of Dentistry, University of British Columbia, Vancouver, BC Canada

**Keywords:** Dental care, Disparities, Disability, Preventive dental care, Psychosocial factors, Social support, Stress, Unmet dental needs

## Abstract

**Background:**

Children and adolescents with special health care needs (SHCN) have higher unmet dental needs, but the potential mechanisms by which parental factors can influence dental care use have not been determined. Parenting a child with SHCN can present special demands that affect parents’ well-being and, in turn, their caregiving. Hence, the study's overall aim was to apply the stress process model to examine the role of parental psychosocial factors in the association between child SHCN and dental care. Specifically, the study tested hypotheses regarding how (a) children’s SHCN status is associated with child dental care (unmet dental needs and lack of preventive dental visits), both directly and indirectly via parental psychosocial factors (parenting stress, instrumental, and emotional social support) and (b) parental social support buffers the association between parenting stress and child dental care.

**Methods:**

A secondary data analysis of the 2011–2012 US National Survey of Children’s Health was performed for 6- to 11-year-old children (n = 27,874) and 12- to 17-year-old adolescents (n = 31,328). Our age-stratified models estimated associations between child SHCN status and parental psychosocial factors with two child dental care outcomes: parent-reported unmet child dental needs and lack of preventive dental care.

**Results:**

Parents of children with (vs without) SHCN reported higher unmet child dental needs, higher parenting stress, and lower social support (instrumental and emotional). Instrumental, but not emotional, parental support was associated with lower odds of their child unmet dental needs in both age groups. The association between parenting stress and child dental care outcomes was modified by parental social support.

**Conclusion:**

Differences existed in child unmet dental needs based on SHCN status, even after adjusting for parental psychosocial factors. SHCN status was indirectly associated with unmet dental needs via parental instrumental support among adolescents, and parental instrumental support buffered the negative association between parenting stress and both child dental care outcomes. Hence, parental social support was an important determinant of child dental care and partially explained the dental care disparities in adolescents with SHCN.

**Supplementary Information:**

The online version contains supplementary material available at 10.1186/s12903-022-02314-y.

## Background

In the United States (US), dental care is the most frequently reported unmet health care need among children with special health care needs (SHCN) [1, 2]—i.e., children who “have or are at increased risk for a chronic physical, developmental, behavioral, or emotional condition and who also require health and related services of a type or amount beyond that required by children generally” [3]. Unmet dental needs are a troubling phenomenon for this population, given that children with SHCN face a range of oral health and oral health care access disparities compared with those without SHCN [4–6]. Furthermore, unmet dental needs have been more frequently reported among children with SHCN who were older, were from low-income families, had poor oral health, and had/or have a severe disability [2, 5, 7, 8]. Since such unmet dental needs are tied to a higher risk of dental diseases, increasing dental care access, including access to preventive dental care, is important for children with SHCN [9].

The provision of dental care services in the US is largely privatized and financed mainly through three sources: private dental insurance (48.6%), out-of-pocket payments (41.6%), and public programs (9.3%) [10]. Public dental care coverage for some children and adolescents with SHCN is provided through Medicaid and/or State Children’s Health Insurance Program (SCHIP) programs. These federal programs provide a range of regulatory and funding mechanisms to the states, some of which require the use of federal allocation to serve eligible children and adolescents with SHCN. Children and adolescents enrolled in Medicaid are entitled to comprehensive oral health services through the Early and Periodic Screening, Diagnosis and Treatment program. Children and adolescents with SHCN in the US can receive professional dental health care in clinics of general dentists and pediatric dentists, as well as dental hygienists. According to the American Academy of Pediatric Dentistry guidelines, children with SHCN should establish a dental home by 12 months of age and a dental recall program based on caries risk, oral health needs and patient capabilities [11].

Higher unmet dental needs in children with (vs. without) SHCN can be attributed to child-, caregiver-, dental-, and health care-related factors, including poor oral self-care and dietary behaviors [12], severity of disability [13], caregiver burden [14], lack of or inadequate health insurance and financial strain [15, 16], shortage of dental professionals with clinical competence in managing patients with SHCN [17], and competing medical care needs for children with SHCN [13]. Furthermore, the effects of parental psychosocial factors on such children’s dental care are critical but understudied. A growing body of literature exists on the broader influence of parental psychosocial factors, such as parenting stress and social support, on children’s oral health [18–21]. However, less is known about how these factors apply to SHCN populations.

Caring for children with SHCN can affect parents’ mental, emotional, and physical well-being, which, in turn, might influence their ability to engage in preventive healthy behaviors for their children, such as seeking dental care. Regardless of age, children with SHCN rely heavily on parents/caregivers as primary decision-makers for their oral health needs. Consequently, the parents of these children are exposed to more stressors and may have access to fewer resources than do other parents [22–24]. For instance, caregiver burden has been associated with lower preventive dental use in children with SHCN aged 3–17 years [25]. However, access to resources such as social support can have a positive effect on both children’s and caregivers’ health [24]. High caregiver social support has been shown to mitigate caregivers’ stress [24] and increase children’s dental care utilization [26]. Caregiver social support can also influence children’s dental care utilization by increasing opportunities for caregivers to access oral health-related information, as well as increasing community and personal resources that may facilitate children’s timely dental visits [19, 27]. Despite the importance of parents’ health and well-being for their children’s (dental) health care, especially those with SHCN [28], few studies have examined whether parental psychosocial factors, such as parenting stress and social support, impact children’s dental care [14, 25]. Evaluating such associations may assist health care providers in enhancing dental care services for children with SHCN.

**Fig. 1:**
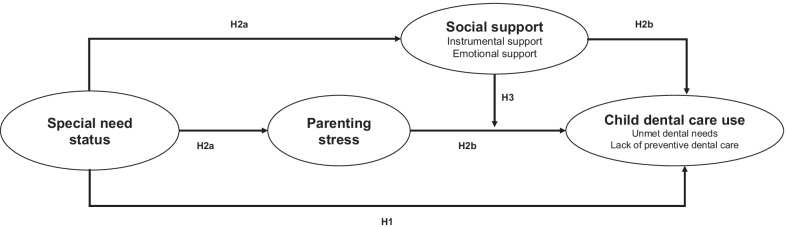
Conceptual model and proposed study hypotheses. (Path numbers correspond to specific hypotheses.)

The present study examines the extent to which parental psychosocial factors may contribute to dental care disparities in children and adolescents with and without SHCN. Our study is based on a conceptual model (Fig. 1) informed by the stress process model [29] to consider the effects of parental caregiving-related stressors and resources on oral health, in turn, this could provide an explanation for SHCN-related oral health disparities. The stress process model is a dominant theoretical framework for examining the mechanisms generating social patterns in exposure to stress and recognizes that exposure to social stressors is fundamentally linked to social statuses and social roles [29, 30]. While the model has been widely used to examine the processes underlying general health, especially mental health, less attention has been given to its application in oral health research [21]. Our model explored the infrequently considered role of psychosocial factors in the study of oral health inequalities in children and adolescents. Stressors are conceptualized as primary (i.e., linked to children with SHCN) and secondary (i.e., linked to caregiving demands) stressors. The association between a child’s SHCN status and parenting stress has been well-documented [22]: having a child with SHCN may generate high levels of parenting stress, which can negatively influence parental attitudes and caretaking responsibilities toward their children, including seeking dental health. Parental resources, such as social support, can facilitate children’s dental care by helping to support the parents and lessen (or buffer) the effects of the stressors. From this model, we formulated and empirically tested three hypotheses with respect to two child oral health outcomes (unmet dental needs and preventive dental visits):

### H1

Children with SHCN are more likely to have higher unmet dental needs (outcome 1) and lack preventive dental visits (outcome 2) than are children without such needs.

### H2

The associations between a child’s SHCN and their dental care-related outcomes are mediated by parental psychosocial factors (parenting stress and social support), such that children with SHCN have higher unmet dental needs and lack preventive dental visits due to higher levels of parenting stress and lower levels of parental social support.

### H3

The negative associations between parenting stress and child dental care-related outcomes are buffered by parental social support.

## Methods

### Data source and study population

We tested these hypotheses using data from the 2011–2012 US National Survey of Children's Health (NSCH), a cross-sectional survey administered by the National Center for Health Statistics of the Centers for Disease Control and Prevention to examine children's health and well-being within family and community contexts. The survey used a random-digit-dialing sampling method across the 50 states and the District of Columbia between February 2011 and June 2012 to identify households with children under 18 years old [31–33]. In each identified household, one child was randomly selected and a parent/guardian living in the household with the most knowledge about the child was interviewed using a computer-assisted telephone interviewing system (70% of the caregivers interviewed were mothers). Hereafter, we refer to any of these adult respondents as "parents”. The current study was exempted from the University of British Columbia Behavioral Research Ethics Board review. The reporting of the study followed the Strengthening the Reporting of Observational Studies in Epidemiology (STROBE) guidelines (Additional file 1: S1 Checklist).

We restricted our analyses to children from 6 to 17 years of age (n = 65,680). Children < 6 years were excluded because determinants of dental care utilization, such as family income [34] and parental psychosocial factors [18], can vary with a child’s age [35]. Also, dental care utilization patterns are usually low for this age group [35, 36]. After restricting our analyses to cases with no missing values for the study variables (90.1%), the final analytical sample included 59,175 children (Additional file 1: Fig. S1).

### Dependent variables

We focused on two dimensions of child dental care, both based on parental reports. Our first outcome, the child’s unmet dental needs, was a binary variable derived from responses to two items: “During the past 12 months, was there any time when your child needed health care, but it was delayed or not received?” and “What type of care was delayed or not received? Mark all that apply: medical care, dental care, vision care, mental care, or something else.” Following an affirmative response to the first item, the second item addressed the type of health care. Children who needed dental care but did not receive it were classified as having unmet dental needs and coded as “1”, with all others coded “0.”

The second outcome, lack of preventive dental visits, was a binary variable based on the response to a single item: “During the past 12 months, how many times did your child see a dentist for preventive dental care such as check-ups and dental cleanings?” Children who did not have a dental visit for preventive care in the past 12 months were coded as “1,” and those with at least one dental visit coded “0.” This coding was based on evidence that an annual dental visit provides needed dental health benefits [37].

### Independent variables

Our key independent variables included child’s SHCN status and three parental psychosocial factors: parenting stress and instrumental and emotional social support.

Child’s SHCN status was determined using a five-item, caregiver-reported screening tool developed by the Child and Adolescent Health Measurement Initiative to identify children with SHCN based on ongoing health consequences rather than on a specific diagnosis or disability [3, 38]. Regardless of the medical diagnosis, a child screened positive for SHCN status if at least one of the following five consequences was reported due to ongoing health conditions that lasted (or expected to last) a minimum of 12 months: (1) need for prescribed medications to manage ongoing medical, behavioral, or other chronic health conditions; (2) need for greater than average use of medical, mental health, or educational services; (3) use of or need for specialized therapies (physical, occupational, or speech therapy); (4) presence of emotional, developmental, or behavioral problems requiring treatment or counseling; or (5) presence of functional age-related limitations as a result of ongoing health conditions [39]. Based on this screener tool, a three-category variable was constructed: (1) non-SHCN (i.e., children who did not qualify for any of the above screening conditions); (2) SHCN without functional limitations (i.e., children who qualified for a prescription medication screener criterion or at least one of the three services listed above but did not qualify as having a functional limitation); and (3) SHCN with functional limitations (i.e., children with one or more age-related functional limitation(s) alone or in combination with other defining criteria).

Parenting stress was assessed from the three-item Aggravation in Parenting Scale [40], with each item having a five-point response (always, usually, sometimes, rarely, never). Parents were asked how often in the past month (1) they felt that their child was much harder to care for compared with other same-aged children, (2) they felt that their child does things that bothered them (the parents) a lot, and (3) they felt angry with their child. The parenting stress score was calculated by averaging the values of all item of the scale (theoretical range = 1–5), with a higher score indicating higher levels of parenting stress (weighted Cronbach’s $\mathrm{\alpha }$ = 0.70).

Instrumental social support was assessed based on the availability of assistance to parents in the neighborhood, such as: (1) “people in my neighborhood help each other out, (2) “we watch out for each other’s children in this neighborhood”, (3) “there are people I can count on in this neighborhood”, and (4) “if my child was outside playing and got hurt or scared, there are adults nearby who I trust to help my child.” All statements had a four-point response, from strongly disagree to strongly agree. These items were initially developed for the US Longitudinal Studies of Child Abuse and Neglect and have been used in many other surveys [33]. The derived score was the average of the four items (theoretical range = 1–4), with a higher score indicating more instrumental support (weighted Cronbach’s $\mathrm{\alpha }$ = 0.86).

Emotional social support was measured by whether parents had someone to whom they could turn to for day-to-day emotional help with parenthood/child-raising [41] (coded as available = 1; unavailable = 0). The weighted correlation between emotional social support and instrumental social support was 0.23, thus we considered them as two distinct subconstructs.

### Control variables

Our analyses controlled for several sociodemographic factors (see Table 1) relevant to dental care use in children and adolescents: child’s age, gender, race/ethnicity, health insurance coverage, family structure, number of children < 18 years of age in the household, perceived neighborhood safety, and family socioeconomic status measured by family income and parental education.

**Table 1:** Characteristics of study sample as reported by parents of children aged 6–17 years—2011/2012 National Survey of Children’s Health

	6–11 years old (%), n = 27,847			12–17 years old (%), n = 31,328		
	Special health care needs (SHCN)			Special health care needs (SHCN)		
	No	Yes, but no functional limitations	Yes, with functional limitations	No	Yes, but no functional limitations	Yes with functional limitations
Subgroup n	21,207	5186	1454	23,186	6366	1776
Dental care variables						
Unmet dental needs	2.7	3.2	4.0	3.3	4.7	9.8
Lack of preventive dental visits	11.8	10.2	13.9	14.4	12.0	16.4
Independent variables						
Parenting stress, mean (SE)^a^	1.9 (0.01)	2.2 (0.03)	2.6 (0.07)	1.9 (0.01)	2.2 (0.02)	2.5 (0.06)
Social support						
Instrumental, mean (SE)^b^	3.4 (0.01)	3.4 (0.02)	3.3 (0.04)	3.4 (0.01)	3.4 (0.02)	3.2 (0.04)
Emotional, % available	90.5	90.1	81.0	88.4	90.0	85.2
Control variables						
*Family income*						
< 100% of FPL	18.5	21.2	26.6	15.9	15.8	25.3
100–199% of FPL	21.3	23.1	22.5	20.3	19.6	20.2
200–399% of FPL	30.5	28.4	33.5	30.6	29.7	26.9
≥ 400% FPL	29.6	27.3	17.4	33.3	34.9	27.6
*Parental education*						
< High school	21.7	15.8	24.3	22.7	16.8	22.7
High school	31.4	36.6	32.6	34.4	33.6	35.8
> High school	46.9	47.6	43.1	42.9	49.6	41.5
*Gender of the child*						
Male	48.2	60.8	63.9	50.4	52.5	58.8
Female	51.8	39.2	36.1	49.6	47.5	41.2
*Race/ethnicity*						
Non-Hispanic white	53.5	59.1	49.3	57.9	63.5	56.5
Non-Hispanic black	12.4	15.3	18.7	13.2	14.8	14.3
Hispanic	23.7	16.6	22.7	20.0	13.3	18.7
Multi-racial/other	10.4	9.0	9.3	9.0	8.3	10.5
*Family structure*						
Two parents	77.5	68.4	62.5	75.1	69.3	63.4
Other family types	22.5	31.6	37.5	24.9	30.7	36.6
*Children in a household*						
One child	15.1	18.3	17.3	26.4	26.9	25.4
2+ children	84.9	81.7	82.7	73.6	73.1	74.6
*Perceived neighborhood safety*						
Never/sometimes	12.9	14.0	20.6	10.6	10.9	17.7
Usually/always	87.1	86.0	79.4	89.4	89.1	82.3
*Health Insurance*						
Private	6.1	3.3	3.2	6.8	2.6	2.9
Public	62.0	58.2	43.1	67.4	64.4	50.7
Uninsured	32.0	38.4	53.7	25.8	33.1	46.3

Percentages/estimates are weighted, reported n’s are unweighted; FPL = federal poverty level; SE = standard error; Percentages are column totals; ^a^Parenting stress scale range = 1–5; ^b^Instrumental social support scale range = 1–4

### Statistical analyses

All analyses were conducted using Stata/SE version 16.1 software (StataCorp LP, 2013), with the svyset feature according to the NSCH guidelines for utilizing probability weights and accounting for the complex sampling design [32]. Descriptive sample characteristics were computed and stratified by SHCN status and age groups. The analyses proceeded separately for children and adolescents using the following steps:

For hypothesis one, we tested for SHCN-related disparities in both dental care outcomes using binary logistic regression models that progressively regressed unmet dental needs or lack of preventive dental visits on (1) SHCN status and (2) SHCN status and parental psychosocial variables—in both cases, adjusting for all control variables.

To test hypothesis two regarding whether SHCN status was indirectly associated with child dental care outcomes via parental psychosocial factors, we assessed whether: (1) SHCN status was associated with the parental psychosocial variables (path a) by regressing each parental psychosocial factor (parenting stress, instrumental social support, and emotional social support) on SHCN status and control variables; and (2) parental psychosocial variables were associated with child dental care outcomes, controlling for SHCN status and control variables (path b, already estimated from the abovementioned second model used to test hypothesis one). The presence of an indirect relationship was determined if (in path a) SHCN was significantly associated with any of the three psychosocial variables and (in path b) any specific psychosocial variable found associated with SHCN in path a was also significantly associated with one or both child dental care outcomes.

Last, for hypothesis three regarding whether parental social support moderated the relationship between parenting stress and each child dental care outcome, we included interaction terms for parenting stress and each type of social support (i.e., the product terms of parenting stress × emotional social support and parenting stress × instrumental social support) in another set of binary logistic regression models, similar to those for hypothesis one.

Because interactions in nonlinear models based on odds ratios can be difficult to interpret [42], we evaluated the interaction using Stata’s margins and marginsplot commands to calculate and graph the predicted probabilities of the two child dentalcare outcomes. For these analyses, we report the predicted probability of a child’s unmet dental needs or lack of preventive dental visits for each level of parenting stress according to specific values of parental social support (i.e., available and unavailable emotional support and the 25th and 75th percentile values of instrumental support) for all interactions between parenting stress and social support, to ease interpretation.

For linear and binary logistic model results, we report unstandardized slope coefficients (b) and adjusted odds ratios (AORs), respectively, with their 95% confidence intervals (CI). Furthermore, to better facilitate comparison of estimates across models, Additional file 1: Tables S1–S3 report all the estimates for these models as average marginal effects (AMEs). An AME is, for a continuous independent variable, the average change in the predicted probability of the outcome for each one-unit increase in the independent variable or, for a categorical variable, the difference in the predicted probability of an outcome between two categories of that variable. A *p* value of < 0.05 was considered statistically significant.

## Results

### Sample characteristics

The final analytic sample included 59,175 children and adolescents, of whom 24% and 26% had SHCN, respectively (Additional file 1: Fig. S2). Table 1 presents the weighted relative frequencies based on SHCN status. Higher proportions of children with SHCN had unmet dental needs compared with their peers without SHCN, especially those with functional limitations (4.0% in 6- to 11-year-olds and 9.8% in 12- to 17-year-olds). Higher rates of lack of preventive dental care visits in the previous 12 months were observed among 12- to 17-year-olds. However, a pattern existed across both age groups, whereby the highest proportions of those not receiving preventive dental visits existed among children with SHCN and functional limitations (13.9% in 6- to 11-year-olds and 16.4% in 12- to 17-year-olds), followed by those without SHCN (11.8% in 6- to 11-year-olds and 14.4% in 12- to 17-year-olds), and then those with SHCN but no functional limitations (10.2% in 6- to 11-year-olds and 12.0% in 12- to 17-year-olds).

For the three psychosocial variables, parents of children with SHCN reported comparatively higher levels of parenting stress, with the highest stress reported by parents of children with SHCN with functional limitations (mean_6–11_ = 2.6 and mean_12–17_ = 2.5). Parental instrumental support was similar across both age groups for all three SHCN subgroups (mean range = 3.2–3.4). However, for emotional support, in both age groups, parents of children with SHCN with functional limitations reported lower levels of emotional support (approximately 81% for ages 6–11 and 85% for ages 12–17) than did parents of children without SHCN.

### Multivariable results: child dentalcare outcomes

Multivariable model results for both age groups are presented in Table 2 (unmet health care needs) and Table 3 (preventive dental visits). The SHCN-related disparities in child dental care existed only among the 12-to 17-year-old subgroup: significant estimates were only found for unmet dental needs, but not lack of preventive dental visits (Model 1). Compared with children with no SHCN, children with SHCN with and without functional limitations had higher odds of unmet dental needs [AOR_12–17_ = 3.17 (95% CI 1.91, 5.26) and AOR_12–17_ = 1.58 (95% CI 1.11, 2.25)]. In both age groups, no significant differences in preventive dental visits among the three SHCN status subgroups.

**Table 2:** Adjusted odds ratios (95% confidence intervals) from binary logistic regression models for parent-reported child unmet dental needs regressed on special health care needs status and parental psychosocial factors—2011/2012 National Survey of Children’s Health

	Model 1	Model 2	Model 3	Model 4	Model 5
*6–11 years old (n = 27,847)*					
*Child special health care needs status*					
No	1.00	1.00	1.00	1.00	1.00
Yes (no functional limitations)	1.20 (0.83, 1.74)	1.15 (0.79, 1.68)	1.18 (0.81, 1.71)	1.20 (0.83, 1.74)	1.15 (0.78, 1.68)
Yes (functional limitations)	1.41 (0.76, 2.60)	1.30 (0.67, 2.51)	1.37 (0.74, 2.52)	1.41 (0.75, 2.62)	1.30 (0.67, 2.53)
*Parental psychosocial factors*					
Parenting stress		1.13 (0.93, 1.37)			1.09 (0.89, 1.33)
Social support					
Instrumental support			0.64*** (0.52, 0.79)		0.64*** (0.52, 0.79)
Emotional support (reference: unavailable)				0.98 (0.61, 1.55)	1.08 (0.69, 1.69)
*12–17 years old (n = 31,328)*					
*Child special health care needs status*					
No	1.00	1.00	1.00	1.00	1.00
Yes (no functional limitations)	1.58* (1.11, 2.25)	1.47* (1.04, 2.09)	1.52* (1.07, 2.16)	1.58* (1.11, 2.25)	1.43* (1.01, 2.03)
Yes (functional limitations)	3.17*** (1.91, 5.26)	2.84*** (1.58, 5.09)	3.02*** (1.82, 5.03)	3.17*** (1.91, 5.27)	2.75*** (1.53, 4.95)
*Parental psychosocial factors*					
Parenting stress		1.22* (1.01, 1.49)			1.18 (0.98, 1.44)
Social support					
Instrumental support			0.66*** (0.54, 0.80)		0.67*** (0.55, 0.81)
Emotional support (reference: unavailable)				0.98 (0.62, 1.53)	1.07 (0.68, 1.68)

*p < 0.05; ** *p* < 0.01; ***p < 0.001

All estimates weighted; Models 1–4 columns report estimates from four separate models, each of which includes a single independent variable (child special health care needs status, parenting stress, instrumental support or emotional support) and all sociodemographic factors (family income, parental education, child age, gender, race/ethnicity, family structure, number of children in household, neighborhood safety, and health insurance); Model 5 column reports estimates from full models that include child special health care needs status, all parental psychosocial factors, and all sociodemographic factors

**Table 3:** Adjusted odds ratios (95% confidence intervals) from binary logistic regression models for lack of child preventive dental visits regressed on special health care needs status and parental psychosocial factors—2011/2012 National Survey of Children’s Health

	Model 1	Model 2	Model 3	Model 4	Model 5
*6–11 years old (n = 27,847)*					
*Child special health care needs status*					
No	1.00	1.00	1.00	1.00	1.00
Yes (no functional limitations)	0.87 (0.67, 1.13)	0.85 (0.65, 1.11)	0.87 (0.67, 1.13)	0.87 (0.67, 1.13)	0.85 (0.65, 1.11)
Yes (functional limitations)	1.13 (0.74, 1.71)	1.07 (0.69, 1.68)	1.12 (0.73, 1.71)	1.12 (0.73, 1.71)	1.06 (0.67,1.68)
*Parental psychosocial factors*					
Parenting stress		1.08 (0.94, 1.23)			1.07 (0.94, 1.22)
Social support					
Instrumental support			0.89 (0.76, 1.03)		0.89 (0.77, 1.04)
Emotional support (reference: unavailable)				0.92 (0.65, 1.29)	0.94 (0.67, 1.33)
*12–17 years old (n = 31,328)*					
*Child special health care needs status*					
No	1.00	1.00	1.00	1.00	1.00
Yes (no functional limitations)	0.92 (0.73, 1.16)	0.91 (0.73, 1.15)	0.91 (0.72, 1.14)	0.92 (0.73, 1.16)	0.91 (0.72, 1.14)
Yes (functional limitations)	1.08 (0.78, 1.50)	1.06 (0.76, 1.48)	1.06 (0.77, 1.47)	1.08 (0.78, 1.50)	1.05 (0.76, 1.47)
*Parental psychosocial factors*					
Parenting stress		1.03 (0.92, 1.14)			1.02 (0.91, 1.13)
Social support					
Instrumental support			0.89 (0.78, 1.02)		0.90 (0.78, 1.03)
Emotional support (reference: unavailable)				0.93 (0.71, 1.22)	0.94 (0.72, 1.24)

*p < 0.05; ** *p* < 0.01; ***p < 0.001

All estimates weighted; Models 1–4 columns report estimates from four separate models, each of which includes a single independent variable (child special health care needs status, parenting stress, instrumental support or emotional support) and all sociodemographic factors (family income, parental education, child age, gender, race/ethnicity, family structure, number of children in household, neighborhood safety, and health insurance); Model 5 column reports estimates from full models that include child special health care needs status, all parental psychosocial factors, and all sociodemographic factors

### Multivariable results: parental psychosocial factors

Table 4 reports associations between child SHCN status and parental psychosocial variables. Compared with parents of children with no SHCN, parents of children with SHCN either with or without functional limitations reported similarly high levels of parenting stress regardless of the child’s age group: for the “SHCN with functional limitations” group, *b*_6–11_ = 0.65 (95% CI 0.51, 0.67) and *b*_12–17_ = 0.58 (95% CI 0.44, 0.71); for the “SHCN without functional limitations” group *b*_6–11_ = 0.34 (95% CI 0.28, 0.39) and *b*_12–17_ = 0.30 (95% CI 0.26, 0.35).

**Table 4:** Adjusted slope coefficients and odds ratios (95% confidence intervals) from linear and binary logistic regression models for the associations between special health care needs status and each parental psychosocial factor—2011/2012 National Survey of Children’s Health

	Parenting stress	Instrumental social support	Emotional social support (available vs. non-available)
	*b* (95% CI)	*b* (95% CI)	AOR (95% CI)
*6–11 years old (n = 27,847)*			
*Special health care needs status*			
No	1.00	1.00	1.00
Yes (no functional limitations)	0.34*** (0.28, 0.39)	− 0.02 (− 0.06, 0.02)	0.93 (0.72, 1.20)
Yes (functional limitations)	0.65*** (0.51, 0.79)	− 0.08 (− 0.16, 0.00)	0.51*** (0.35, 0.75)
*12–17 years old (n = 31,328)*			
*Special health care needs status*			
No	1.00	1.00	1.00
Yes (no functional limitations)	0.30*** (0.26, 0.35)	− 0.07** (− 0.12, − 0.03)	1.13 (0.90, 1.41)
Yes (functional limitations)	0.58*** (0.44, 0.71)	− 0.13** (− 0.20, − 0.05)	0.92 (0.65, 1.30)

*p < 0.05; ** *p* < 0.01; ***p < 0.001

All estimates weighted; *b* = regression slope coefficient; AOR = adjusted odds ratio; CI = confidence interval. All models are adjusted for family income, parental education, child age, gender, race/ethnicity, family structure, number of children in household, neighborhood safety, and health insurance

For instrumental social support, although parents of children with (vs without) SHCN in both age groups reported lower levels, only parents in the 12- to 17-year-old group had significant estimates (i.e., *b*_12–17_ =  − 0.13 and − 0.07, respectively, for SHCN with and without functional limitations). Similar findings existed for emotional support, but with a different age pattern: parents of children with any SHCN also had lower levels of available emotional support, though only the estimates for the 6- to 11-year-old group with functional limitations (vs non-SHCN) were significant [AOR_6–11_ = 0.51 (95% CI 0.35, 0.75)].

### Parental psychosocial factors and dentalcare outcomes

Models 2–5 in Tables 2 and 3 summarize results for the independent associations between each parental psychosocial factor and child dental care outcomes, net of SHCN status, and sociodemographic factors. Overall, none of the three parental psychosocial factors was significantly associated with child preventive dental visits in either age group. We discuss the specific findings in terms of each psychosocial variable across models that tested the variable’s association with the outcome before and after controlling for the other psychosocial variables.

For parenting stress, Table 2, Model 2, indicates that higher stress levels were only associated with higher odds of child unmet dental needs in the 12- to 17-year-old group (AOR_12–17_ = 1.22; 95% CI 1.01, 1.49). This initial association loses statistical significance in Model 5, which adjusts for the two social support variables.

For social support, Model 3 for both age groups (Tables 2, 3) indicates that higher parental instrumental support was independently associated with lower odds of unmet child dental needs [AOR_6–11_ = 0.64 (95% CI 0.52, 0.79); AOR_12–17_ = 0.66 (95% CI 0.55, 0.82)]. In the fully adjusted models (Model 5), instrumental support remained inversely and significantly associated with the outcome [AOR_6–11_ = 0.64 (95% CI 0.52, 0.79); AOR_12–17_ = 0.67 (95% CI 0.55, 0.81)]. Emotional support was not significantly associated with this outcome in Models 4 or 5.

Consistent with our hypothesis two (grounded in the stress process model), we evaluated the potential mediating role of parental psychosocial factors in shaping dental care disparities in SHCN for any pathways where estimates for SHCN status → psychosocial variable (path a, Table 4) and each psychosocial variable → child dental care outcomes, net of SHCN status (path b, Tables 2, 3) were significant. Additional file 1: Table S4 summarizes associations to identify potential mediation according to these criteria. The statistically significant estimates observed in the 12- to 17-year-old group support the mediating role of the stress process factors. Specifically, children who have SHCN with and without functional limitations (vs non-SHCN) were each indirectly associated with higher odds of unmet dental needs via the psychosocial pathway of lower instrumental parental social support. No evidence of mediation of parental psychosocial factors was observed in the 6- to 11-year-old group.

### Social support as a buffer of parenting stress and child dental care outcomes

**Fig. 2:**
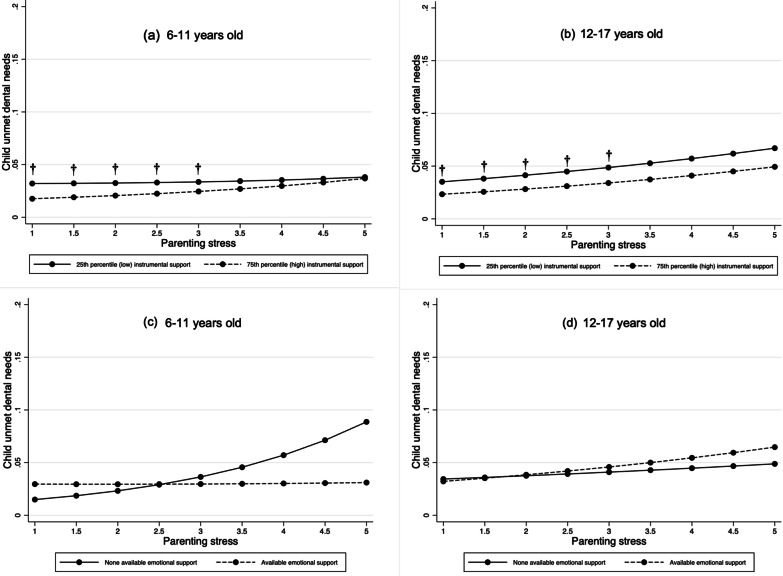
Predicted probabilities for child unmet dental needs and parenting stress by parental social support level. *Note*: Results shown are based on models reported in Additional file 1: Table S5; † indicates that, at a specific level of parenting stress, the predicted probabilities of child unmet dental needs for each level of support are significantly different (*p* < 0.05) based on estimated average marginal effects. Because instrumental support is a continuous variable, only results for the 25th and 75th percentile values are shown for ease of visualization. However, the corresponding statistical significance tests were based on all values of instrumental support—not just the difference between those two percentiles—for each specific level of parenting stress

**Fig. 3:**
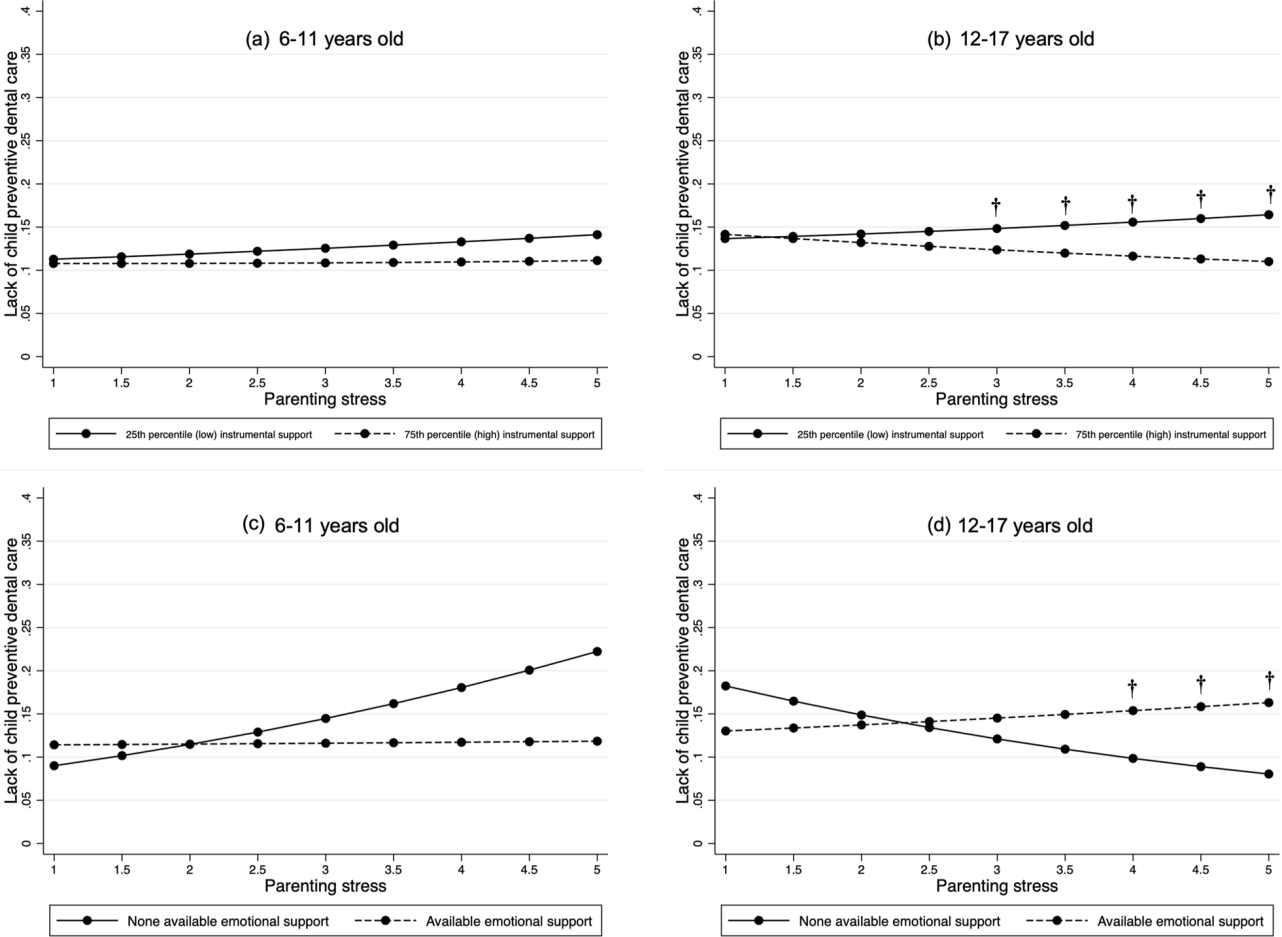
Predicted probabilities for lack of child preventive dental visits and parenting stress by parental social support level. *Note*: Results shown are based on models reported in Additional file 1: Table S5; † indicates that, at a specific level of parenting stress, the predicted probabilities of child unmet dental needs for each level of support are significantly different (*p* < 0.05) based on estimated average marginal effects. Because instrumental support is a continuous variable, only results for the 25th and 75th percentile values are shown for ease of visualization. However, the corresponding statistical significance tests were based on all values of instrumental support—not just the difference between those two percentiles—for each specific level of parenting stress

To test hypothesis three, we evaluated the potential buffering effect of instrumental and emotional social support on the relationship between parenting stress and the two dental care outcomes. Figures 2 and 3 plot the results from our regression models that tested this buffering hypothesis via introducing interaction terms for parenting stress and parental social support into the Model 5 in Tables 2 and 3. Specific model estimates for these interaction terms and other key variables are reported in Additional file 1: Table S5, and supplementary analyses (not shown) testing these interactions separately (vs. all in the same model) found similar results.

Each graph displays the predicted probabilities of one of the two outcomes for a specific age group, at specific values of parenting stress and instrumental and emotional social support. This enables a clearer interpretation than model estimates. For the instrumental support graphs, results are displayed for only the 25th and 75th percentile values of support to facilitate interpretation of a continuous variable. However, any statistical significance noted in the graph for instrumental support is based on an AME (i.e., the average change in the probability of the outcome for each one-unit increase in instrumental support at a specific level of stress) that was computed based on all empirical values of instrumental support and was statistically significant (*p* < 0.05). By contrast, for each graph showing emotional support (a binary variable), tests of statistical significance were based on AME that tested the difference between the predicted probability of the outcome for each of the two emotional support categories (available/unavailable) at each specific level of stress.

In graphing all interaction results and testing for significant differences in the predicted probabilities of the outcome (via estimating AME), we only found partial support for the buffer hypothesis of parenting stress.

For instrumental support, the associations between parenting stress and higher likelihoods of (a) child unmet dental needs and (b) no preventive dental visits were significantly weaker in both age groups among parents with higher levels of support. As shown in Fig. 2, Panels a and b, the predicted probability of child unmet dental needs among those with high (at the 75th percentile) vs low (at the 25th percentile) instrumental support was reduced by 1.0–2.0 percentage points (pp) for parents with lower levels of parenting stress (i.e., scores between 1 and 3) in both age groups. However, those differences are substantively small and were not observed for higher levels of parenting stress. By contrast, for lack of preventive dental visits among 12- to 17-year-olds (Fig. 3, Panel b), there was a 3.0–6.0 pp difference in the probability of this outcome among parents with high (75th percentile) vs low (25th percentile) instrumental support at higher levels of parenting stress (i.e., scores between 3 and 5).

A similar buffering pattern was observed in the 6-to 11-year-old group for parents reporting available emotional support and higher stress levels, but the differences in the predicted probabilities for both outcomes were statistically non-significant (Figs. 2, 3, Panel c). For parents with the highest reported stress (i.e., score = 5) the probabilities of child unmet dental needs and lack of preventive dental visits were 6.0 pp (*p* = 0.132) and 10.0 pp (*p* < 0.10) lower, respectively, among those reporting available (vs. unavailable) access to emotional support.

However, for the 12- to 17-year-old group (Fig. 3, Panel d), a reverse pattern was observed. Among parents reporting the highest stress scores of 4 or 5, the probability of them not taking their child to preventive dental visits was 8.0 pp (*p* < 0.007) lower among parents without available emotional support vs those with such support.

## Discussion

We began this study concerned with understanding dental care needs among children with SHCN. Guided by the stress process model, we formulated a priori hypotheses regarding how differences in parental psychosocial factors may uniquely contribute to patterns of dental care among children with and without SHCN. In analyzing US national data, we found some empirical support for our hypotheses as well as findings not in accordance with those shown in previous research. Below, we discuss our results in terms of the study hypotheses.

### Child special health care needs and child dental care

Partially corroborating our hypotheses, SHCN-based disparities in child dental care were limited to unmet dental needs (not preventive dental visits)—and mainly among the 12- to 17-year-olds—among whom children with SHCN (with and without functional limitations) had higher odds of unmet dental needs.

The importance of having dental access for children with SHCN should not be underestimated. As an indicator of poor access to dental care, unmet dental needs can most likely result in poor oral health as these children age [43]. As children with SHCN transition into adolescence and young adulthood, they may experience additional challenges to establishing appropriate individual dental care, including accessing the health care system [44]. The extent of unmet dental needs will also worsen over time, which will make appropriate oral health care management challenging for both parents and dental providers. Consistent with prior research [2, 45], we found a significant association between unmet dental needs and SHCN status in adolescents. This could potentially be related to parents' lack of information about oral health and dental clinics and services available for this age group [46, 47], lack of proper communication between the parents and dental team [47], and dental professional limited knowledge and skills related to treating this population [17]. In addition, some parents of children with SHCN may be unaware of dental services available to them, which puts their children at a higher risk of unmet dental needs. Parents of children with SHCN have expressed a need for multidisciplinary collaboration and care coordination to better address their children’s dental needs [48].

In examining two dimensions of dental care—unmet dental needs and lack of preventive dental visits—we expected that children and adolescents with SHCN would use less preventive dental care due to their high medical needs, which might supplant their parents’ concerns for seeking preventive dental visits [49], and/or their parents’ choice of seeking urgent care over preventive dental care [50]. This can be more apparent in children with SHCN who have more complex health needs since previous research has shown that children with severe chronic conditions [13] and/or functional limitations [49] were less likely to use preventive dental care. Also, it is important to considering that having access to dental care that accommodates children with SHCN might depend on the availability and geographical distribution of dental professionals having the knowledge and skills to care for children with SHCN [51]. However, we observed no differences in preventive dental visits based on SHCN status. Our findings contribute to existing evidence that has been contradictory, which might be due to variations in the study samples (e.g., age) and outcomes studied. For instance, Van Cleave and Davis [4] found that only younger children (aged 3–5 years old) with (vs without) SHCN were more likely to attend a preventive dental visit. Lida et al. [49] found that children with (vs without) SHCN were more likely to only attend non-preventive dental visits (e.g., restorations, extraction). In addition, our findings reflect US children and adolescents with SHCN and may not be representative of other countries having a public dental health care coverage for children and adolescents.

### Parental psychosocial factors and dental care in children with SHCN

Informed by the stress process model, our second hypothesis focused on (a) whether SHCN differences existed in regards to parental psychosocial factors and (b) whether such parental psychosocial factors were associated with child dental care. Our findings partially supported the role of stress process factors, since SHCN was indirectly associated with unmet dental needs via instrumental parental support in adolescents. Many complex processes may influence child and adolescent oral health, and the stress process model can provide a useful tool to identify the processes by which stressors and resources can influence oral health and explain some of the potential pathways leading to oral health inequalities.

For the first component of this hypothesis, parents of children with (vs. without) SHCN experienced higher parenting stress and lower social support. This finding is consistent with those of previous studies [22] reporting that families of children with SHCN face additional burdens and higher parenting stress because of the complexity of their child’s health condition, the demand to coordinate multiple visits to health providers, and the challenges of daily home-based care. Collectively, these duties may disrupt parents’ ability to address their child’s overall health needs, which can make dental care less prioritized [14, 25]. Our findings suggest that parental psychosocial factors and their childcare responsibilities may be influenced by their child’s SHCN. Therefore, utilizing medical (and dental) homes to help facilitate and coordinate the care needed for parents can subsequently mitigate the psychosocial risk factors for children with SHCN and their families [24].

For the second component of this hypothesis—that parental psychosocial factors were associated with child dentalcare outcomes—we found partial support: only lower parental instrumental support was associated with unmet dental needs. Our findings suggest that this may be a mediating pathway between a child’s SHCN status and unmet dental needs.

Although parents of children with SHCN experienced higher parenting stress, contrary to our hypothesis, such stress was not associated with child dental care for either age group. Previous studies have reported that parenting stress was associated with increased [18] and decreased [25] child dental visits, while others have found no such association [20, 21]. Several possible explanations are possible. First, these studies are not directly comparable with ours because they examined different outcomes and/or mediators. This is important because, within the stress process model, the relationships among stressors, resources, and outcomes can be context-specific. For instance, Masterson and Sabbah [20] found a positive association between maternal stress, indicated by allostatic load, and child dental caries. Second, most of the studies on access to dental care have not distinguished between primary (preventive) and secondary/tertiary (treatment- or urgency-based) dental care. Possibly, stressed parents are preoccupied, which makes them pay more attention to their child’s dental pain and problems compared with following preventive oral health measures for their children. Third, our sample included children both with and without SHCN, and this differed from Nelson et al.’s [18] focus on children without SHCN and Chi et al.’s [25] focus on children with SHCN.

Similar to our findings, prior evidence has suggested that lower parental social support is much more common among parents of children with SHCN [52] and has been linked to poor child oral health outcomes and reduced child dental care [19, 27, 41, 53]. However, unlike our study, no previous studies have examined all three domains—special needs status, parental psychosocial factors, and dental care outcomes—and their interrelationships.

Our findings provide insights into the role of parental social support in the dental care of children with SHCN. Parenting children with SHCN can limit parents’ social interactions and increase social isolation, which might cause parents to miss opportunities to access oral health knowledge and share social norms. In turn, this can lead to reduced adherence to healthy behaviors such as oral self-care and regular dental attendance for children. Previous work [47] has suggested that providing parents of children with SHCN with support and tools prior to the dental visit can improve not only child attendance but also involvement during the dental clinic. This is in line with Imms and colleagues' work [54], which identified parental support as a key extrinsic factor that facilitates children's participation [52].

For our third and final hypothesis, we expected that parents with high social support would be better adapted to parenting stress, and thus positively affecting their child’s dental care. Consistent with this stress-buffering hypothesis, our findings suggest that parental social support offers some protection against the adverse effect of parenting stress on child dental care. Thus, social support may mitigate the negative influence of parenting stress on child dental care by, for example, providing an outlet for parents to discuss problems and share responsibilities, and facilitating parental well-being and effective coping skills [52]. But for parents with low social support, parenting stress may have a substantially negative effect on their child’s dental care.

## Limitations and strengths

Several study limitations should be noted. First, though we used a unique nationally representative US dataset (NSCH) that included children and adolescents with SHCN, which allowed us to compare those with and without SHCN [55], the cross-sectional study design of NSCH limits causal inferences. Future prospective cohort studies are needed to examine the relationships between parental psychosocial factors and dental care for children and adolescents with SHCN. Nevertheless, our data allowed us to look at a more comprehensive model to explain dental care in populations with SHCN, upon which future research can further build.

Second, the NSCH relied on parental reports of child dental care needs and utilization that may be subject to bias. For example, parental reports might inaccurately reflect their children’s true unmet needs [56]. However, parental reports provide complementary insights and are usually considered the main driver for seeking dental care.

Third, our preventive dental visits outcome was based on one question that might not have adequately distinguished between preventive (primary prevention) and treatment-based (secondary prevention) dental visits. Future validation work should consider linking self-reports with administrative data.

Fourth, our three-item parenting stress scale assessed important aspects of stress and has been psychometrically evaluated [40]. However, it might be limited in capturing all the stressors that parents could experience.

Finally, our analysis did not account for a number of parental and family factors that may contribute to child dental care. Future studies should examine parents’ dental behaviors, notably their oral self-care and dental visits patterns. In addition, our model did not include measures from all domains in the stress process model, a limitation that could be addressed in future research (e.g., using mixed-methods approaches).

Despite these limitations, this study provides new evidence that parental psychosocial factors—especially low social support—may exert effects on dental care–seeking behaviors in parents of children with SHCN. Since optimal oral health care continues to be challenging among this population [57], it is beneficial for dental and public health researchers to collaborate and better understand how to leverage the role of parents and families to ensure greater uptake of dental services by populations with SHCN [24, 28].

## Recommendation for future research

Our findings highlight the need to involve various stakeholders such as policymakers, dental providers, and families to improve dental health services to address the dental needs of children and adolescents with SHCN. Health services may consider the importance of psychosocial screening and care coordination for parents/caregivers of children with SHCN, which might lead to better overall health for both children and their families [24]. Age-specific intervention strategies may be needed to support parents and their children. Some examples include educating parents about importance of oral care and diet to child oral health and helping parents navigate the dental health care system to identify dental providers who treat children and adolescents with SHCN. Parents should also know that their children with SHCN are in greater need of regular preventive dental care due to their increased risk of dental disease [58, 59]. In addition, dental providers need to be trained in treating patients with SHCN, especially those with functional limitations [5, 56, 60].

## Conclusion

In conclusion, we found differences in unmet dental needs based on child SHCN status, even after adjusting for parental psychosocial factors. Child SHCN status was indirectly associated with unmet dental needs via parental instrumental social support in adolescents, and parental instrumental social support buffered some of the negative associations between parenting stress and unmet child dental needs.

## Supplementary Information

**Additional file 1.** Supporting Figures (S1 and S2) and Tables (S1–S5).

## Data Availability

The NSCH dataset analyzed in the current study is available from the Centers for Disease Control and Prevention National Center for Health Statistics database: https://www.cdc.gov/nchs/slaits/nsch.htm#anchor_1551498813689

## Abbreviations

US: United States
SHCN: Special Health Care Needs
NSCH: National Survey of Children’s Health
FPL: Federal poverty level
SE: Standard error
AOR: Adjusted odds ratio
CI: Confidence interval
pp: Percentage points
