# Supplementary material for: Parental psychosocial factors, unmet dental needs and preventive dental care in children and adolescents with special health care needs: A stress process model
Source: BMC Oral Health. 2022 Jul 11;22:282. doi: 10.1186/s12903-022-02314-y (PMC9275152; doi:10.1186/s12903-022-02314-y)
Supplement: Supplementary file 1 — Additional file 1. Supporting Figures (S1 and S2) and Tables (S1–S5). [file 12903_2022_2314_MOESM1_ESM.pdf]

Supporting information

Parental psychosocial factors unmet dental needs and preventive dental care in children and adolescents with special health-care needs: A stress process model.

Arwa Z. Gazzaz, Richard M. Carpiano, Denise M. Laronde, Jolanta Aleksejuniene

S1 Figure. Flowchart of study sample

S2 Figure. Proportions of children and adolescents according to special health-care needs status

S1 Table. Average marginal effects (AMEs) and 95% confidence intervals (CIs) from binary logistic regression models for parent-reported child unmet dental needs — 2011/2012 National Survey of Children's Health.

S2 Table. Average marginal effects (AMEs) and 95% confidence intervals (CIs) from binary logistic regression models for parent-reported child lack of preventive dental visits — 2011/2012 National Survey of Children's Health.

S3 Table. Average marginal effects (AMEs) and 95% confidence intervals (CIs) for parental psychosocial factors — 2011/2012 National Survey of Children's Health.

S4 Table. Summary of observed associations between special health-care need status; parental psychosocial factors; and child dental-care outcomes variables.

S5 Table. Adjusted odd ratios (95% confidence intervals) from binary logistic regression models for parent-reported child unmet dental needs and lack of preventive dental visits regressed on special health-care need status and psychosocial parental factors with interaction — 2011/2012 National Survey of Children's Health

S1 Checklist. STROBE statement—checklist of items that should be included in reports of cross-sectional observational studies.

S1 File. Stata commands.

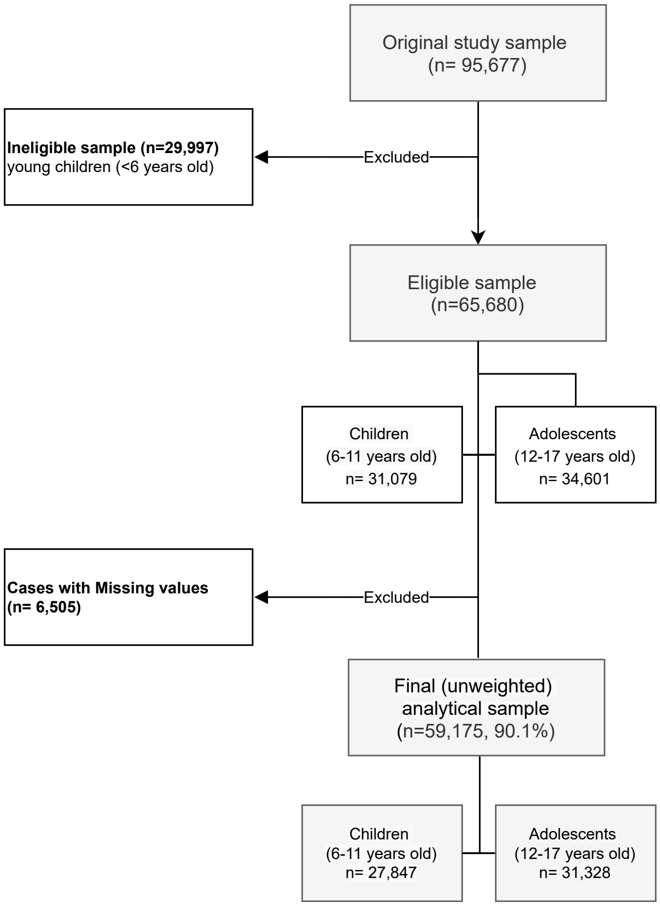

Figure S1. Flowchart of study sample

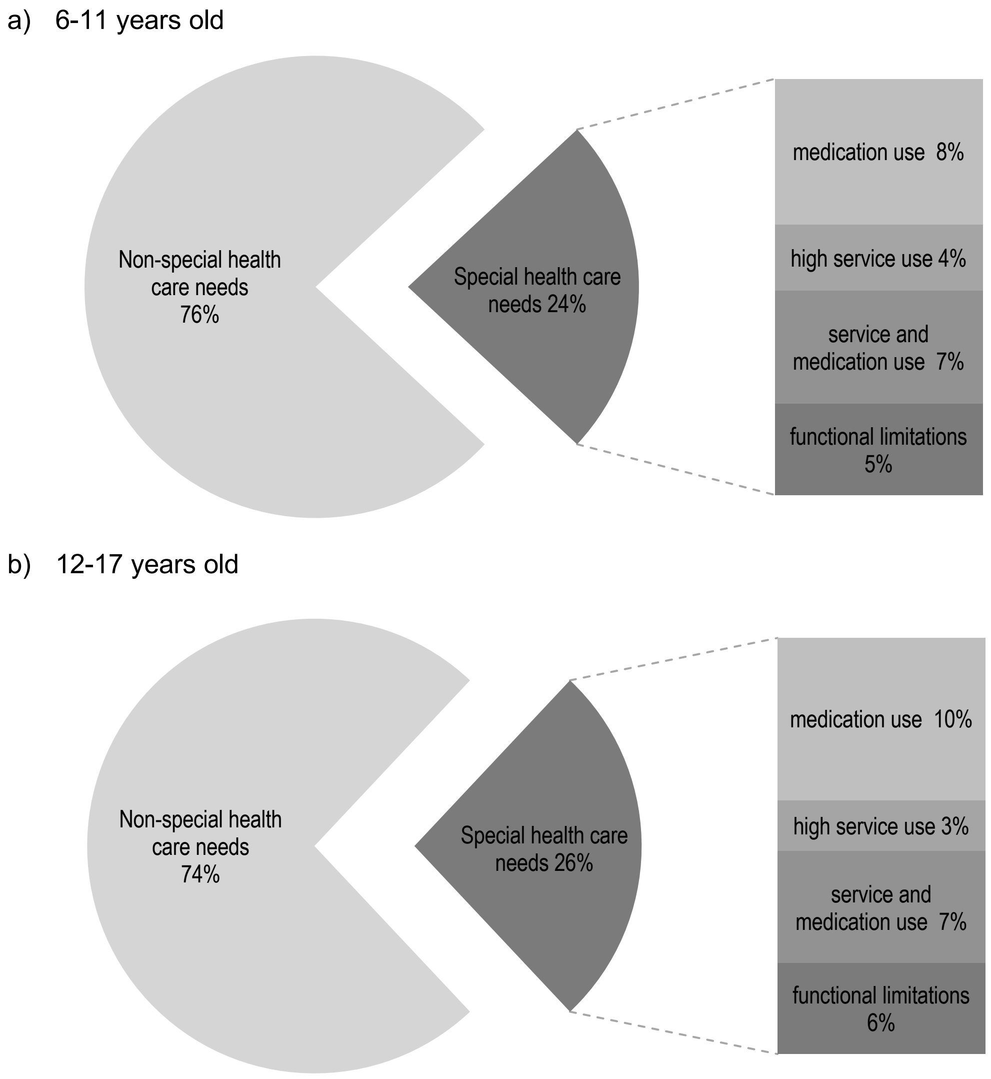

Figure S2. Proportions of children and adolescents according to special health-care needs status

Table S1. Average marginal effects (AMEs) and 95% confidence intervals (CIs) of child unmet dental needs regressed on special health-care need status and parental psychosocial factors — 2011/2012 National Survey of Children's Health

**6-11 years old (n= 27,847)**	**Model 1**	**Model 2**	**Model 3**	**Model 4**	**Model 5**
**Child special health-care need status**					
No	Referent	Referent	Referent	Referent	Referent
Yes (no functional limitations)	0.01<br>(-0.01, 0.02)	0.00<br>(-0.01, 0.01)	0.00<br>(-0.01, 0.02)	0.01<br>(-0.01, 0.02)	0.00<br>(-0.01, 0.01)
Yes (functional limitations)	0.01<br>(-0.01, 0.03)	0.01<br>(-0.01, 0.03)	0.01<br>(-0.01, 0.03)	0.01<br>(-0.01, 0.03)	0.01<br>(-0.01, 0.03)
**Parental psychosocial factors**					
Parenting stress		0.00<br>(-0.00, 0.01)			0.00<br>(-0.00, 0.01)
Social support					
Instrumental support			-0.01***<br>(-0.02, -0.01)		-0.01***<br>(-0.02, -0.01)
Emotional support (referent=unavailable)				-0.00<br>(-0.01, 0.01)	0.00<br>(-0.01, 0.01)
**12-17 years old (n=31,328)**	**Model 1**	**Model 2**	**Model 3**	**Model 4**	**Model 5**
**Child special health-care need status**					
No	Referent	Referent	Referent	Referent	Referent
Yes (no functional limitations)	0.02*<br>(0.00, 0.03)	0.01<br>(-0.00, 0.02)	0.02*<br>(0.00, 0.03)	0.02*<br>(0.00, 0.03)	0.01<br>(-0.00, 0.03)
Yes (functional limitations)	0.06**<br>(0.02, 0.10)	0.05*<br>(0.01, 0.09)	0.06**<br>(0.02, 0.09)	0.06**<br>(0.02, 0.10)	0.05*<br>(0.01, 0.09)
**Parental psychosocial factors**					
Parenting stress		0.01*<br>(0.00,0.01)			0.01<br>(-0.00, 0.01)
Social support					
Instrumental support			-0.01***<br>(-0.02, -0.01)		-0.01***<br>(-0.02, -0.01)
Emotional support (referent=unavailable)				-0.00<br>(-0.02, 0.02)	0.00<br>(-0.01, 0.02)

*$p<0.05$; ** $p<0.01$; ***$p<0.001$.

Note. Results obtained from binary logistic regression models. All estimates weighted; All models adjust for sociodemographic factors (family income, parental education, child age, gender, race-ethnicity, family structure, number of children in household, neighborhood safety and health insurance).

Table S2. Average marginal effects (AMEs) and 95% confidence intervals (CIs) of child lack of preventive dental visits regressed on special health-care need status and parental psychosocial factors — 2011/2012 National Survey of Children's Health

**6-11 years old (n= 27,847)**	**Model 1**	**Model 2**	**Model 3**	**Model 4**	**Model 5**
**Child special health-care need status**					
No	Referent	Referent	Referent	Referent	Referent
Yes (no functional limitations)	-0.01	-0.01	-0.01		-0.01
	(-0.04, 0.01)	(-0.04, 0.01)	(-0.04, 0.01)		(-0.04, 0.01)
Yes (functional limitations)	0.01	0.01	0.01		0.01
	(-0.03, 0.06)	(-0.04, 0.05)	(-0.03, 0.05)		(-0.04, 0.05)
**Parental psychosocial factors**					
Parenting stress		0.01			0.01
		(-0.01, 0.02)			(-0.01, 0.02)
Social support			-0.01		-0.01
			(-0.03, 0.00)		(-0.03, 0.00)
Instrumental support					
Emotional support				-0.01	-0.01
(referent=unavailable)				(-0.04, 0.03)	(-0.04, 0.03)
**12-17 years old (n= 31,328)**	**Model 1**	**Model 2**	**Model 3**	**Model 4**	**Model 5**
**Child special health-care need status**					
No	Referent	Referent	Referent	Referent	Referent
Yes (no functional limitations)	-0.01	-0.01	-0.01	-0.01	-0.01
	(-0.03, 0.01)	(-0.03, 0.01)	(-0.03, 0.01)	(-0.03, 0.02)	(-0.03, 0.02)
Yes (functional limitations)	0.01	0.01	0.01	0.01	0.01
	(-0.03, 0.04)	(-0.03, 0.04)	(-0.03, 0.04)	(-0.03, 0.04)	(-0.03, 0.04)
**Parental psychosocial factors**					
Parenting stress		0.00			0.00
		(-0.01, 0.01)			(-0.01, 0.01)
Social support					
Instrumental support			-0.01		-0.01
			(-0.03, 0.00)		(-0.03, 0.00)
Emotional support					-0.01
(referent=unavailable)					(-0.04, 0.02)

*p<0.05; ** p<0.01; ***p<0.001.

Note. Results obtained from binary logistic regression models. All estimates weighted; All models adjust for sociodemographic factors (family income, parental education, child age, gender, race-ethnicity, family structure, number of children in household, neighborhood safety and health insurance).

Table S3. Average marginal effects (AMEs) and 95% confidence intervals (CIs) for each parental psychosocial factors regressed on child special health-care need status— 2011/2012 National Survey of Children's Health.

	**Parenting stress** [a]	**Instrumental social support**[a]	**Available (vs unavailable) Emotional social support** [b]
**6-11 years old (n= 27,847)**	AME (95% CI)	AME (95% CI)	AME (95% CI)
**Special health-care need status**			
No	Referent	Referent	Referent
Yes (no functional limitations)	0.34*** (0.28, 0.39)	-0.02 (-0.06, 0.02)	-0.01 (-0.03, 0.02)
Yes (functional limitations)	0.65*** (0.51, 0.79)	-0.08 (-0.16, 0.00)	-0.07** (-0.11, -0.02)
	**Parenting stress** [a]	**Instrumental social support** [a]	**Available (vs unavailable) Emotional social support** [b]
**12-17 years old (n= 31,328)**	AME (95% CI)	AME (95% CI)	AME (95% CI)
**Special health-care need status**			
No	Referent	Referent	Referent
Yes (no functional limitations)	0.30*** (0.26, 0.35)	-0.07** (-0.12, -0.03)	0.01 (-0.01, 0.03)
Yes (functional limitations)	0.58*** (0.44, 0.71)	-0.13** (-0.20, -0.05)	-0.01 (-0.04, 0.03)

**p*<0.05; ** *p*<0.01; ****p*<0.001. Results obtained from [a] linear regression and [b] binary logistic regression models. All estimates weighted; All models are adjusted for family income, parental education, child age, gender, race-ethnicity, family structure, number of children in household, neighborhood safety and health insurance..

Table S4. Summary of observed associations between special health-care need status; parental psychosocial factors; and child dental-care outcomes variables.

6-11 years old (n= 27,847)			
**Special health-care need status**	**Special health-care need status → Parental psychosocial factor**	**Parental psychosocial factor → Child dental-care outcomes**	
		**Unmet dental needs**	**Lack of preventive dental visits**
No (reference)			
Yes - no functional limitations	Parenting stress (+)	(NS)	(NS)
	Instrumental social support (NS)	(-)	(NS)
	Emotional social support (NS)	(NS)	(NS)
Yes - functional limitations	Parenting stress (+)	(NS)	(NS)
	Instrumental social support (NS)	(-)	(NS)
	Emotional social support (-)	(NS)	(NS)
**12-17 years old (n= 31,328)**			
**Special health-care need status**	**Special health-care need status → Parental psychosocial factor**	**Parental psychosocial factor → Child dental-care outcomes**	
		**Unmet dental needs**	**Lack of preventive dental visits**
No (reference)			
Yes - no functional limitations	Parenting stress (+)	(NS)	(NS)
	Instrumental social support (-)	(-)	(NS)
	Emotional social support (NA)	(NS)	(NS)
Yes - functional limitations	Parenting stress (+)	(NS)	(NS)
	Instrumental social support (-)	(-)	(NS)
	Emotional social support (NS)	(NS)	(NS)

Note. Reference category for special health-care need status = non-special health-care needs; The symbols (+) and (−) respectively indicate positive and negative associations between either (a) a special health-care need variable and parental psychosocial variable or (b) a parental psychosocial variable and child dental-care outcomes. All such notation indicates an association where the 95% confidence interval (CI) did not include 0 (for a linear model) or 1 (for a non-linear model). (NS) indicate that the 95% CI for the observed associations included either 0 for a linear model or 1 for a non-linear model.

Table S5. Adjusted odd ratios (95% confidence intervals) from binary logistic regression models for parent-reported child unmet dental needs and lack of preventive dental visits regressed on child special health-care need status and psychosocial parental factors with interaction — 2011/2012 National Survey of Children's Health

	**Unmet dental needs**	**Lack of preventive dental visits**
	Model 6	Model 6
**6-11 years old (n= 27,847)**	AOR (95% CI)	AOR (95% CI)
**Child special health-care need status**		
No	1.00	1.00
Yes (without functional limitations)	1.13 (0.77, 1.65)	0.85 (0.65, 1.11)
Yes (with functional limitations)	1.25 (0.64, 2.46)	1.05 (0.67, 1.66)
**Parental psychosocial factors**		
Parenting stress	1.01 (0.54, 1.89)	1.64 (0.95, 2.83)
Social support		
Instrumental support	0.46** (0.28, 0.76)	1.01 (0.70, 1.46)
Emotional support (ref. unavailable)	3.30** (1.35, 8.07)	1.76 (0.88, 3.51)
**Interaction**		
Parenting stress x instrumental support	1.16 (0.95, 1.42)	0.94 (0.80, 1.11)
Parenting stress x emotional support	0.62** (0.44, 0.89)	0.76 (0.56, 1.02)
	**Unmet dental needs**	**Lack of preventive dental visits**
	Model 6	Model 6
**12-17 years old (n= 31,328)**	AOR (95% CI)	AOR (95% CI)
**Child special health-care need status**		
No	1.00	1.00
Yes (without functional limitations)	1.44 * (1.02, 2.03)	0.90 (0.72, 1.12)
Yes (with functional limitations)	2.75 *** (1.52, 5.00)	1.04 (0.75, 1.45)
**Parental psychosocial factors**		
Parenting stress	1.02 (0.56, 1.87)	1.21 (0.80, 1.83)
Social support		
Instrumental support	0.63 (0.40, 1.01)	1.21 (0.89, 1.64)
Emotional support (ref. unavailable)	0.85 (0.29, 2.45)	0.45* (0.23, 0.88)
**Interaction**		
Parenting stress x instrumental support	1.02 (0.85, 1.24)	0.87* (0.76, 0.98)
Parenting stress x emotional support	1.10 (0.76, 1.60)	1.42* (1.08, 1.86)

*p<0.05; ** p<0.01; ***p<0.001.

Note. All estimates weighted; Model 6 column reports estimates from models adjusted for special health-care needs, parental psychosocial factors, and all sociodemographic characteristics (family income, parental education, child age, gender, race-ethnicity, family structure, number of children in household, neighborhood safety and health insurance) and interactions between parenting stress and social support variables; AOR=adjusted odds ratio; CI=confidence interval.
